# Liver microphysiological platforms for drug metabolism applications

**DOI:** 10.1111/cpr.13099

**Published:** 2021-07-22

**Authors:** Gulsim Kulsharova, Akbota Kurmangaliyeva

**Affiliations:** ^1^ School of Engineering and Digital Sciences Nazarbayev University Nur‐Sultan Kazakhstan; ^2^ School of Sciences and Humanities Nazarbayev University Nur‐Sultan Kazakhstan

**Keywords:** microfluidics, drug development, drug metabolism, liver‐on‐a‐chip, microphysiological platforms, multi‐organ chips

## Abstract

Drug development is a costly and lengthy process with low success rates. To improve the efficiency of drug development, there has been an increasing need in developing alternative methods able to eliminate toxic compounds early in the drug development pipeline. Drug metabolism plays a key role in determining the efficacy of a drug and its potential side effects. Since drug metabolism occurs mainly in the liver, liver cell‐based alternative engineering platforms have been growing in the last decade. Microphysiological liver cell‐based systems called liver‐on‐a‐chip platforms can better recapitulate the environment for human liver cells in laboratory settings and have the potential to reduce the number of animal models used in drug development by predicting the response of the liver to a drug in vitro. In this review, we discuss the liver microphysiological platforms from the perspective of drug metabolism studies. We highlight the stand‐alone liver‐on‐a‐chip platforms and multi‐organ systems integrating liver‐on‐a‐chip devices used for drug metabolism mimicry in vitro and review the state‐of‐the‐art platforms reported in the last few years. With the development of more robust and reproducible liver cell‐based microphysiological platforms, the drug development field has the potential of reducing the costs and lengths associated with currently existing drug testing methods.

## Graphical abstract

Liver‐on‐a‐chip (LOC) devices are microfluidic platforms seeded with human liver cells able to recapitulate the functions, microphysiology, and drug metabolism that takes place in the human liver. Liver‐on‐a‐chip platforms in stand‐alone format and coupled to other microphysiological devices (multi‐organ platforms) can open doors towards accelerating drug development and reducing the number of animal models used in the future.

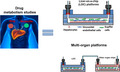

## 1 INTRODUCTION

Drug development is a highly inefficient process with failures of many drugs due to toxicity or lack of efficacy. On average, it takes around 10 years for a new pharmaceutical compound to enter the market and costs approximately US $2.6 billion.^1^ The majority of drug failures in clinical trials occur due to the low capability of existing preclinical models to predict the toxicity of compounds.^2^ Although the majority of potential drug candidates entering drug development are identified during initial in vitro stages of drug metabolism investigations and drug discovery,^3^ a high portion of the compounds producing toxic metabolites are not identified early enough. Some compounds deemed to be successful in in vitro drug metabolism studies perform well on animal models but fail in the last stages of clinical trials.^4^, ^5^ There continues to be a critical need to enhance our understanding of drug metabolism and to help eliminate toxic compounds early in the drug development pipeline.

Liver cells play a major role in drug metabolism studies in drug development. Conventional and emerging in vitro methods used in metabolism studies are based on mimicking the action of drug metabolizing enzymes (DMEs) (an important central group of liver proteins).^6^ DMEs are divided into two major groups of phase I and phase II metabolizing enzymes.^7^ Phase I enzymes (Cytochrome P450 and flavin‐containing monooxygenases (FMOs)) serve to make drug compounds more soluble as the majority of drugs come in a lipophilic form. These enzymes are involved in the metabolism of the vast majority of pharmaceuticals (~75% of all marketed drugs).^8^ After a drug goes through a phase I enzyme‐catalysed conversion, its metabolite enters the phase II drug metabolism cycle driven by one of the phase II enzymes (UDP‐dependent glucuronosyltransferases (UGT), sulfotransferases (SULTs), *N*‐acetyltransferases (NATs) and glutathione *S*‐transferases (GSTs)). Phase II enzymes increase the solubility of drug metabolites in water and facilitate their subsequent excretion. In contrast to their phase I counterparts, phase II enzymes are used and studied less in drug testing assays, because they catalyse a smaller range of substrates.

Predicting drug metabolism outcomes driven by the liver enzymes and toxicity of a pharmaceutical or a chemical using improved cell‐based methods has attracted much attention. Currently, in vivo bioactivity of drugs is predicted using physiologically based pharmacokinetic animal models.^9^, ^10^, ^11^ However, a poor correlation between animal data and human outcomes has been observed due to the substantial species‐specific differences in drug metabolism pathways, pharmacokinetics and toxicity targets.^12^, ^13^ Additionally, due to the cost and ethical concerns over animal models, in vitro cultured cells have widely been used as an alternative to animal models,^14^, ^15^ and as a screening tool before the in vivo models.^16^ However, in vitro cell models have major limitations such as tedious sample preparations for separating interfering matrix compounds (proteins, lipids, salts and endogenous compounds) and low accuracy results. Additionally, they are often limited in representing cell function and physiology accurately, which leads to an insufficient translation of preclinical tests to clinical trials.

Microphysiological systems such as organ‐on‐a‐chip platforms have been proposed as a new generation of in vitro models for drug candidate screening in the preclinical phase of drug development.^17^ These cell‐based 3D platforms with microchannels, fabricated using engineering techniques such as microfluidics, are seeded with human organ cells. By allowing the dynamic flow of cell media over cell environment, these platforms aim to mimic closely the anatomy, physiology and functionality of a human organ and can allow improved drug metabolism study outcome.

Among the organ‐on‐a‐chip platforms, liver‐on‐a‐chip (LOC) and LOCs coupled with other organ‐on‐a‐chip (multi‐organ) platforms have emerged due to their potential to better predict in vivo response to the pharmaceutical compounds. The early identification of toxic metabolites using these platforms could cut down the costs of drug testing and reduce the number of animal models.

The number of LOC devices with varying complexities and various applications (liver disease modelling, liver functionality studies, drug screening) is growing every year. Consequently, in recent years, several comprehensive reviews have outlined LOC chip device fabrication and advances in research and market in stand‐alone reviews^18^, ^19^, ^20^ and as part of reviews on microfluidic chips emulating different human organs (organs‐on‐a‐chip).^21^, ^22^, ^23^ However, reported reviews have focused on a wide range of applications of LOC platforms and various aspects. In this contribution, we review LOC platforms and multi‐organ systems developed over the past few years that allow understanding of drug‐induced metabolic responses relevant to the acceleration of drug development. We particularly focus on those platforms employed for the assessment of drug hepatotoxicity, prediction of liver injury induced by drug‐drug interactions, as well as the multi‐organ platforms studying the liver‐other organ crosstalk and the effects of liver metabolism on drug efficacy and toxicity for organ systems.

## 2 LIVER‐ON‐A‐CHIP (LOC) PLATFORMS

Liver is the most important organ in drug metabolism, being a target for drug‐ and chemical‐induced toxicity.^17^ Since the majority of drug metabolism occurs in the liver, most in vitro ADME (adsorption, distribution, metabolism, excretion) and toxicity testing are based on liver cells. Additionally, the liver plays an important role in carbohydrate, protein and lipid metabolism, the synthesis and secretion of blood proteins, and the detoxification of blood.^18^

Perfused microphysiological platforms such as liver‐on‐a‐chip (LOC) platforms have attracted wide attention as an alternative to traditional in vitro models. Various microfluidic LOC systems have been developed to emulate human metabolism using diverse microfabrication techniques based on hepatocytes,^24^, ^25^, ^26^ rat or human liver microsomes,^27^ and embedded human tissue samples.^28^ Using engineering techniques such as microfluidics, the applied medium flow can be controlled very carefully and can mimic blood flow in vessels.^29^ Additionally, the composition of the medium can easily be altered during the course of an experiment, and more physiological medium‐to‐cell ratios can be achieved due to small system volumes.^30^ Moreover, metabolites can reach higher concentrations due to small system volumes of microchannels rendering online detection easier. This is in contrast to metabolites produced in well‐plate experiments where metabolites can be substantially diluted due to the larger well volumes. In microphysiological systems, the metabolite‐containing medium can be directed to other cells located elsewhere on the same chip. To model a correctly functional liver in an in vitro microphysiological system, biotransformation enzymes must be expressed that allow for studying the parent drug and its metabolite production. LOC platforms aim to mimic closely the anatomy, physiology and functionality of the liver using various sources of biotransformation enzymes. Therefore, by combining microfluidics with tissue engineering, the complexity of organ architecture and drug metabolism in vivo can be better mimicked.^31^, ^32^

The LOC systems can be integrated into laboratories as a form of preclinical testing that better predicts human experimentation in vitro.^33^ The chips normally contain either single cultures of hepatocytes, two‐dimensional or 3D co‐cultures of hepatocytes with several other hepatic non‐parenchymal or other stromal cells, hepatocyte spheroids, or organoids formed by mono‐ or co‐cultures and intact liver slices.^17^, ^34^ Furthermore, they allow automated analytics such as monitoring of pH, temperature, waste removal, nutrient supply, fluid pressures and shear stress in culture compartments.^35^

## 3 PLATFORMS FOR DRUG TOXICITY ASSESSMENT/DRUG‐DRUG INTERACTION (DDI) ANALYSIS

The liver plays a principal role in the digestion, metabolism and detoxification of xenobiotic compounds.^36^ Drug‐induced liver injury (DILI), which leads to liver failure and drug attrition,^37^ is the major cause of the withdrawal of approved drug compounds from the market.^38^ Therefore, the need for developing an in vitro model with higher reproducibility of in vivo liver environment is staggering, and microfluidic liver‐on‐a‐chip (LOC) platforms have gained popularity in the research in the domain of drug toxicity assessment.

**FIGURE 1:**
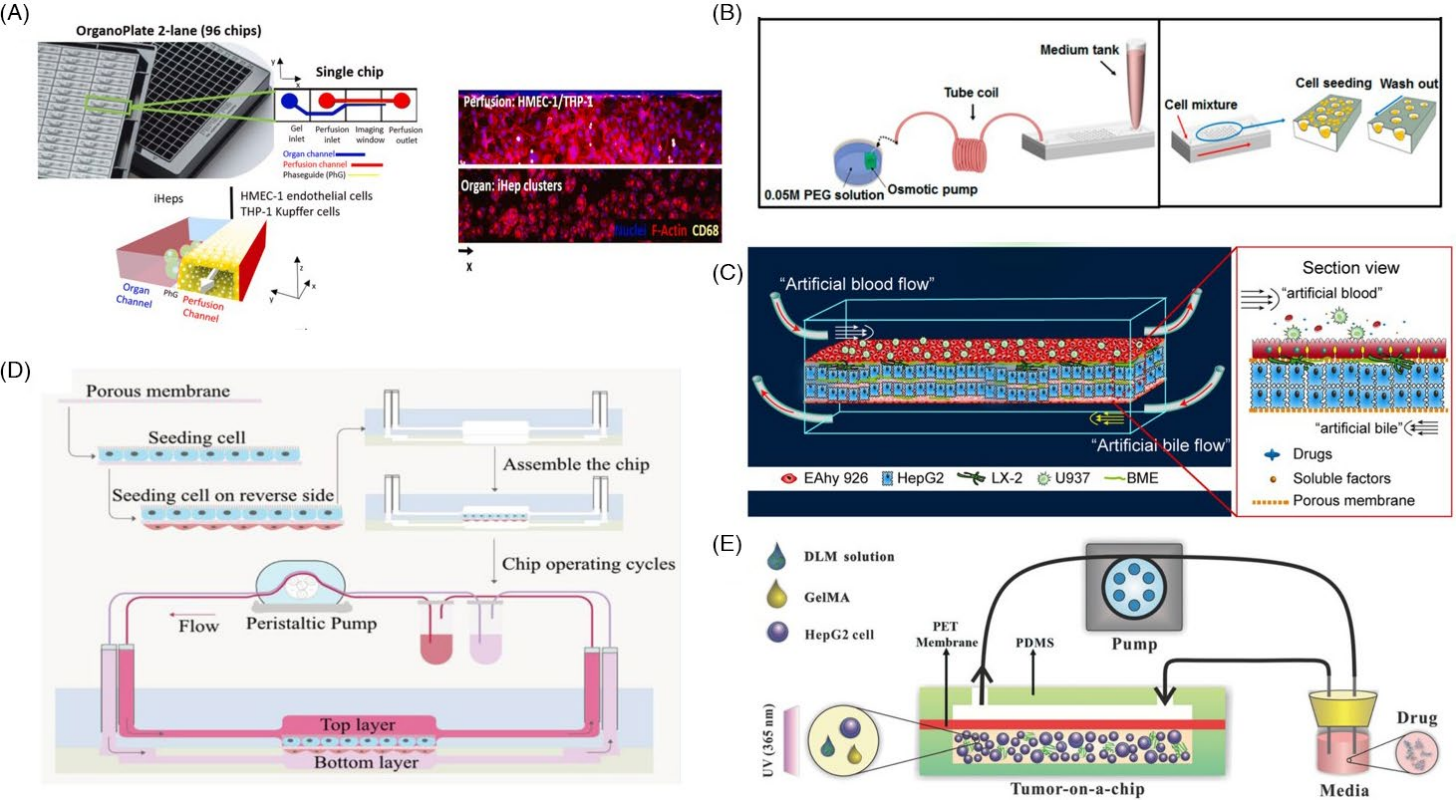
Liver‐on‐a‐chip platforms for drug toxicity assessment/drug‐drug interaction (DDI) analysis (A) OrganoPlate LiverTox culture set‐up and cell culture. Reprinted from Ref. [38], under the terms of the Creative Commons CC‐BY licence. Copyright 2021. (B) Concave microwell array poly‐dimethylsiloxane (PDMS) plates seeded with cells. Reproduced from Ref. [^36^], under the terms of the Creative Commons Attribution License. Copyright 2020. (C) Schematic diagram of 4 kinds of cell lines seeded in the LSOC microdevice. Reproduced from Ref. [41], with permission from AIP Publishing. Copyright 2019. (D) Illustration of the multi‐organ‐on‐a‐chip. From top to bottom, each layer contains Caco‐2, HUVEC, HepG2 and HK‐2, respectively. Reprinted from Ref. [44], Copyright 2020, with permission from Elsevier. (E) Schematic of the DLM‐based liver tumour‐on‐a‐chip and its application for drug toxicity testing. Reproduced from Ref. [48], with permission of The Royal Society of Chemistry

One of the ultimate uses of liver‐on‐a‐chip (LOC) platforms is the high throughput screening of pharmaceutical compounds. In recent years, there have been several state‐of‐the‐art platforms used for the application, which are summarized in Table 1. For example, Bircsak et al., employed the OrganoPlate LiverTox platform (Figure 1A) in its pilot screening to test 159 drug compounds with known hepatotoxicity and rate them according to Toxicological Prioritization values.^38^ The platform has the potential to predict immune‐mediated hepatotoxicity by co‐culturing iPSC‐derived hepatocytes with THP‐1 Kupffer‐like immune cells and HMEC‐1 endothelial cells (Figure 1A).

**TABLE 1:** State‐of‐the‐art liver‐on‐a‐chip platforms used for drug metabolism studies

Model platform	Cells	Drug compound	Duration	Sensitivity	Functions	Ref
OrganoPlate LiverTox™^a^	iHeps, HMEC‐1, THP‐1 Kupffer cells	159 compounds of known hepatotoxicity, troglitazone	15 d	Troglitazone TC_50_ via albumin assay: 57.2 μmol/L TC_50_ values for 159 compounds are provided in the source cited	Development, automation and pilot screening of the high throughput platform Screening and ranking drug compounds by Toxicological Prioritization score Dose‐response evaluation of compounds with known hepatotoxicity Potential for detection of immune‐mediated hepatotoxicity	^38^
Human, rat, dog Liver‐Chip (Emulate Inc), dual‐cell and quadruple‐cell^a^	Dual‐cell: human, rat, dog primary hepatocytes, LSECs Quadruple‐cell: human, rat, dog primary hepatocytes, LSECs, Kupffer cells, stellate cells	Bosentan, APAP, MTX, Janssen proprietary compounds (JNJ‐2, JNJ‐3), TAK‐875	14 d	Bosentan IC_50_: Human: 10 μmol/L Dog: 30 μmol/L Rat: >100 μmol/L	Assessment of human relevance of DILI observed in animal subjects Detection of various DILI phenotypes by means of microscopy, staining and DILI biomarkers Identifying mechanistic end points and biomarkers to clarify DILI mechanisms Assessment of harbinger phenotypes of idiosyncratic DILI	^37^
Liver‐Chip (Emulate Inc)^a^	Primary human hepatocytes, primary LSECs	Diglycolic acid	3 d	Sensitivity values for various conditions are provided in the source cited	Assessment of the overall performance and concordance with in vivo and in vitro studies of Liver‐Chip platform in drug safety and toxicity testing	^39^
Gravity‐driven microfluidic system featuring 3D microtissues (InSphero AG)^a^	Primary hLiMTs, HCT116 TuMTs	CP + RTV IFF + RTV	14 d	–	Detection of altered drug efficacies of CP and IFF upon co‐administration with RTV Application of a user‐friendly and scalable system with a potential to be implemented in industrial settings	^40^
Spheroid‐based 3D artificial liver chip (ALC) with a perfusion function with concave microwell arrays	Rat primary hepatocyte and HSC spheroids	APAP, INH	14 d	Heterospheres APAP IC_50_ Day 3—2.23 μmol/L; Day 7—2.11 μmol/L; Day 14—2.15 μmol/L; INH IC_50_; Day 3—2.96 μmol/L; Day 7—2.81 μmol/L; Day 14—2.66 μmol/L	Application of concave flow and HSC co‐culture to improve predictability of hepatotoxicity screening and metabolic function studies	^36^
‘Homunculus’ Liver‐on‐a‐chip device	HepaRG spheroids	Adaptaquin and analogues	24 h	LC_50_: 600 ± 300 μmol/L	Application of LOC platform to investigate toxicity and metabolism of adaptaquin and analogues Identification of CYP P450 isoform responsible for oxidation of branched tail oxyquinolines	^43^
Various single‐organ and multi‐organ microfluidic chips	HepG2 (LOC), Caco‐2, HUVEC, HK‐2	Ginsenosides compound K (CK) (20‐O‐β‐(D‐glucopyranosyl)‐20(S)‐ protopanaxadiol)	24, 48, and 72 h	–	Pharmacological investigation of a ginsenosides compound K Validation of the reliability of organ‐on‐a‐chip for pharmacological studies	^44^
3D Liver sinusoid‐on‐a ‐chip (LSOC)	HepG2, LX‐2, EAhy926, U937	APAP, RIF, AMI APAP + RIF, APAP + OME, APAP + CPFX	8 d	APAP TC_50_: 9.8 mmol/L RIF TC_50_: 1153.4 μmol/L AMI TC_50_: 315.1 μmol/L	Cell line‐based model producing results comparable with primary hepatocytes in hepatotoxicity, drug‐drug interaction studies Emulate blood and bile flow	^41^
Perfused microscale 3D liver bioreactor (Stem Cell Systems)	Primary human liver cells (not purified of non‐parenchymal cells)	APAP	6 d	–	Validation of the suitability of 3D liver bioreactor for hepatotoxicity studies in perfusion conditions	^45^
Liver‐Chip (Emulate Inc)^a^	Primary human hepatocytes, LSECs	APAP, FIAU	10 d	APAP EC_50_ by albumin assay: 5.6 mmol/L FIAU EC_50_ by albumin assay: 126 μmol/L	Assessment of the sensitivity of Liver‐Chip model and 3D hepatic spheroids to known hepatotoxins (APAP and FIAU)	^46^
Biomimetic three‐dimensional liver tumour‐on‐a‐chip	Based on decellularized liver matrix, HepG2t	APAP Sorafenib	21 d	–	Improved imitation of native tumour microenvironment for pharmacological studies	^48^

Note

– Data not provided.

Abbreviations: AMI, amiodarone; APAP, acetaminophen; CP, cyclophosphamide; CPFX, ciprofloxacin; CYP450, cytochrome P450; DILI, drug‐induced liver injury; EC_50_, half maximal effective concentration; FIAU, fialuridine; hLiMTs, human liver microtissues; HMEC‐1, human microvascular endothelial cells; HSCs, hepatic stellate cells; HUVEC, human umbilical vein endothelial cells; IC_50_, half maximal inhibitory concentration; IFF, ifosfamide; iHeps, iPSC‐derived hepatocytes; INH, isoniazid; LC_50_, lethal concentration 50%; LSECs, liver sinusoidal endothelial cells; MTX, methotrexate; OME, omeprazole; RIF, rifampicin; RTV, ritonavir; TC_50_, median toxic concentration; TuMTs, tumour microtissues.

^a^ Commercially available platforms.

A handful number of LOC platforms have been applied to investigate drug toxicity and DILI. For example, Jang et al began to investigate the mechanisms of an inflammatory response in immune‐mediated DILI in susceptible individuals as well. This ability of the platforms to take the immune system into account may increase concordance between initial hepatotoxicity findings and observed clinical toxicity. Moreover, Jang et al.^37^ propose to use Liver‐Chip in investigating the human relevance of drug toxicity responses in animals by conducting parallel tests on several species‐specific platforms. Food and Drug Administration (FDA) in partnership with a company Emulate investigated the concordance of the Liver‐Chip platforms with in vivo and in vitro studies in drug toxicity testing.^39^ For this purpose, a hepatotoxin diglycolic acid (DGA) was used, and the concordance was assessed in terms of cell viability *via* observing cell morphology, LDH and caspase 3/7 assays, as well as hepatotoxicity by monitoring albumin and urea levels. As a result, the platform provided more physiologically valid data due to the presence of the flow and survival signals from LSECs. However, some morphological features seen in in vivo studies could not be reproduced, and it was concluded that the inclusion of other non‐parenchymal cell types (Kupffer cells, stellate cells) would allow a more accurate representation of responses to drug toxicity.^39^

Another important aspect to be covered by drug metabolism studies are drug‐drug interactions (DDIs) that occur due to modulation of one drug's ADME properties by co‐administration of another drug compound.^40^ Lohasz et al. employed a gravity‐driven microfluidic system with 3D microtissues to analyse DDIs between anticancer prodrugs cyclophosphamide and ifosfamide with antiretroviral ritonavir. Due to the major role of hepatic metabolism in DDIs, the system incorporated human liver microtissues (hLiMTs) in addition to tumour microtissues (TuMTs) which represent a drug target. DDIs were confirmed by metabolite concentration measurements, as well as measurements of TuMTs size and diameter. The study concludes that the described system can be used in early preclinical predictions of DDIs and altered to incorporate various tissues.^40^

Among the studies employing primary hepatocytes for drug metabolism studies is the work done by Choi and colleagues, who propose flow‐based concave microwell arrays to co‐culture primary rat hepatocytes and hepatic stellate cells (HSCs) (Figure 1B). The heterospheres formed as a result of a co‐culture demonstrated higher CYP activity than hepatospheres upon treatment with acetaminophen, and the experiments with repeated exposure of the spheroids to acetaminophen and isoniazid show more stable IC_50_ values in heterospheres. These results suggest that the system is an optimized tool for drug toxicity studies, and due to improved metabolic competence is suitable for investigating drug‐drug interactions (DDIs).^36^

## 4 LIVER‐ON‐A‐CHIP PLATFORMS BASED ON IMMORTALIZED CELL LINES

Cell source is an important aspect in the development of liver‐on‐a‐chip (LOC) platforms used for drug metabolism applications. In general, primary hepatocytes (PH) remain to be the gold standard for the prediction of drug toxicity responses. However, while primary hepatocytes can provide accurate results due to their physiological relevance, there are problems with their extraction, donor‐to‐donor variability, and de‐differentiation, which negatively affect the reproducibility of the results.^41^, ^42^ In the development of microphysiological platforms researchers often prefer immortalized cell lines due to their stability and ease of handling.

In comparison with primary cells, cell lines have lower functionality and weaker reflection of the susceptibility of human liver cells to injury caused by drug toxicity and interactions.^41^, ^42^ Deng *et al*., however, through careful construction of a 3D liver sinusoid‐on‐a‐chip (LSOC) incorporating four immortalized cell lines (HepG2, LX‐2, EAhy926, U937), were able to propose an alternative to primary hepatocyte‐based models with comparable results in testing hepatotoxicity caused by drug‐drug interactions (Figure 1C).

LOC systems employing immortalized cell lines (eg HepG2, Huh7, HepaRG), notwithstanding their less powerful functionality, are very valuable for early‐stage drug development due to their high proliferation and extensive characterization.^42^ LOC platforms seeded with immortalized cell lines are being used in research settings to conduct toxicity and metabolism studies.^39^, ^43^, ^44^ For example, the ‘Homunculus’ LOC device based on HepaRG spheroids was employed to test HIF prolyl hydroxylase inhibitors adaptaquins that show promising results in in vivo haemorrhagic stroke models. Cytochrome P450 (CYP450) enzyme isoforms responsible for compound metabolism were identified, and the study confirmed the suitability of the optimized adaptaquin analogs tested for further preclinical studies.^44^ Liu et al. conducted a pharmacological investigation of carbohydrate‐based drug ginsenoside compound K (CK) using single as well as coupled or multi‐organ chips (Figure 1D). After liver chip experiments, it was revealed that against the hypothesis that CK is mainly metabolized by intestinal microbiota, the liver could also contribute to the process. These results could be attributed to the flow conditions, under which HepG2 cells were able to metabolize CK more readily. Also, the dynamic and more physiologically valid conditions in these microphysiological systems increased cell tolerance to CK, suggesting that in vivo toxicity may be milder than in in vitro static cultures.^44^

Overall, the potential of liver‐on‐a‐chip platforms in drug safety and toxicity testing was reinforced by several studies.^45^, ^46^, ^47^ Corrado et al. developed a microfluidized liver system featuring HepG2 microtissue precursors, which were selected over HepG2 spheroids in the process of development. Following the assessment of the detoxification potential of the platform by ethanol‐induced toxicity tests, this platform was deemed applicable for alcoholic disease mechanisms research, drug toxicity studies and integration into multi‐organ MPSs.^47^ Another study by Freyer et al. proposed the use of a microscale 3D liver bioreactor for hepatotoxicity studies based on an extensive investigation of the dose‐dependent effects of acetaminophen administration into the system. Among the tested parameters were lactate production, ammonia release, levels of inflammatory factors (PGE2, IL‐6), as well as histological and immunohistochemical evaluation and expression of genes for relevant CYPs.^45^ The authors, however, highlight the importance of the inclusion of liver non‐parenchymal cells in correct proportions to increase the sensitivity for drug toxicity.^45^ Foster et al.^46^ present an extensive assessment of a Liver‐Chip system for use in drug toxicity studies. The study investigated the platform's sensitivity to known hepatotoxic drugs acetaminophen (APAP) and fialuridine (FIAU) by employing various functionality (albumin) and injury (α‐GST and miR‐122) biomarkers, as well as drug metabolite quantification. Moreover, the CYP activity profile of the platform was created, and based on all data obtained, the utility of Liver‐Chip models for improved hepatotoxicity risk assessment was confirmed.

Apart from imitating healthy liver physiology, Lu et al. propose to use a model emulating liver tumour microenvironment (TME) for pharmacological studies (Figure 1E). The platform is a biomimetic 3D liver tumour‐on‐a‐chip, which is based on a decellularized liver matrix (DLM) integrated with gelatin methacryloyl (GelMA) and HepG2 cells.^48^ This platform allows for improved imitation of the liver tumour microenvironment and biophysical cues due to the presence of important ECM components from DLM, which is beneficial for more accurate results in drug toxicity studies.^48^ The study tested both the gold‐standard acetaminophen and anticancer model drug sorafenib. Being an imitation of the TME, the platform would especially allow for pharmacological testing of anticancer drugs. Additionally, novel hepatoma cell line‐based models, such as the LSOC proposed by Deng and colleagues, have the potential to compensate for the limitations of cell lines and to serve as an alternative to primary hepatocyte‐based models in drug toxicity studies.^41^

## 5 MULTI‐ORGAN PLATFORMS INTEGRATING LOC

While liver‐on‐a‐chip (LOC) platforms are a promising tool for drug toxicity studies, their integration into coupled or multi‐organ‐on‐a‐chip or body‐on‐a‐chip systems would allow predicting physiological responses to drug administration even more precisely by recapitulating organ‐to‐organ interaction. Multi‐organ‐on‐a‐chip systems would emulate physiological states and drug effects modulated by multi‐organ crosstalk.^20^ Moreover, this technology allows for the investigation of the off‐target toxicity of pharmaceutical compounds and the identification of toxic by‐products of drug metabolism by the liver. State‐of‐the‐art multi‐organ platforms integrated with LOC used for drug metabolism applications are also summarized in Table 2.

**TABLE 2:** Multi‐organ platforms integrating liver‐on‐a‐chip (LOC) used for drug toxicity studies

Organs/platforms	Cells	Compound	Functions	Ref
Liver‐kidney chip	HepG2 Primary glomerular endothelial cells (GECs)	Ifosfamide (IFO) Verapamil (VER)	Evaluation of drug metabolism in liver and subsequent nephrotoxicity	^49^
Microfluidic chip‐based liver‐proximal tubule co‐culture model Microphysiological 2‐OC (TissUse GmbH)^a^	HepaRG, human proximal tubule cell line RPTEC/TERT1	Cyclosporine A (CsA) CsA + rifampicin (RIF)	Mimic the effect of drug‐drug interactions (DDIs) on hepato‐ and nephrotoxicity Potential to elucidate DDIs, drug metabolism and toxicity in combination with detecting the morphology, histopathology and noninvasive toxicity biomarkers	^50^
Heart‐liver multi‐organ microfluidic device	Human primary hepatocytes hiPSC‐derived cardiomyocytes	Cyclophosphamide (CP) Terfenadine (TER)	Evaluation of hepato‐ and cardiotoxicity of drugs and their metabolites by imitating in vivo crosstalk between heart and liver	^51^
Liver‐heart organoids‐ on‐chip	hiPSCs	Clomipramine	Assessment of cardiotoxicity/safety of antidepressant drugs following liver metabolism Emulate organ‐specific functions and drug responses at the multi‐organ level	^52^
Lung/liver‐on‐a‐chip	HepaRG spheroids NHBE cells	Aflatoxin B1 (AFB1)	Ability to assess potential toxicity, as well as hepatotoxicity, of inhaled compounds, and the effect of metabolized compounds on lung Potential application for testing therapeutic compounds	^53^
HUMIMIC Chip3plus—3D air‐liquid interface bronchial model and liver spheroids co‐culture (TissUse GmbH)^a^	Bronchial MucilAir culture Liver spheroids: HepaRG, primary HHSTeC	Aflatoxin B1 (AFB1)	Toxicity assessment of inhaled substances in a microphysiological system allowing organ crosstalk	^54^
Intestine/Liver Two‐Organ‐Chip Microphysiological System (2‐OC MPS) (TissUse GmbH)^a^	Caco‐2 Human colon epithelial cells, HT‐29 human colon carcinoma cells, HepaRG, HHSTeC	Acetaminophen (APAP)	Assessment of pharmacokinetic properties of acetaminophen in microphysiological system	^57^
Intestine‐Liver‐On‐Chip (InLiver‐OC)	H‐InMyoFib, human intestinal epithelial cells Caco‐2, HepG2	Ethanol	Emulation of a first‐pass metabolism of orally administered drugs and chemicals in vitro Demonstration of the intestinal prevention of liver injury and synergistic contribution of intestine and liver tissue models to the release of metabolic enzymes upon ethanol administration	^55^
Pumpless reconfigurable multi‐organ‐on‐a‐chip	Primary human hepatocytes MEG‐01 megakaryocytes, Kasumi‐1 myeloblasts MCF‐7 breast cancer‐derived cells, SW‐962 vulva carcinoma‐derived cells iPSC‐derived cardiomyocytes	Diclofenac, imatinib, tamoxifen, tamoxifen + verapamil	Assessment of on‐target efficacy and off‐target toxicity of anticancer drugs and their metabolites	^58^
Body‐on‐a‐chip (lung, liver, breast cancer) multi‐organ microfluidic platform with breathable lung chamber	A549 (lung), HepG2 C3A (liver), and MDA MB231 (breast cancer)	Curcumin	Comparing inhalation and intravenous drug delivery in terms of effectiveness and toxicity	^59^
Human‐on‐a‐chip system, 7 microphysiological systems—brain, pancreas, liver, lung, heart, gut, endometrium (DARPA Microphysiological Systems Program)	Liver: Cryopreserved human primary hepatocytes, Kupffer cells Gut: C2BBe1, HT29‐MTX‐E2, dendritic cells from frozen Leukopak aliquots Lung: NHBE cells Endometrium: human endometrial epithelial adenocarcinoma cells, tHESCs Brain: NPCs derived from the human H1 ES cell line Heart: Human iCell Cardiomyocytes 2 Pancreas: Rat pancreatic islets	Tolcapone	Tolcapone metabolite profiling and metabolomics Emulation of complex physiological conditions and multi‐organ interactions by combining drug metabolism, metabolomics and cell engineering	60, 61
Various single‐organ and multi‐organ microfluidic chips	HepG2 (LOC), Caco‐2, HUVEC, HK‐2	Ginsenosides compound K (CK) (20‐O‐β‐(D‐glucopyranosyl)‐20(S)‐protopanaxadiol)	Pharmacological investigation of a ginsenosides compound K Validation of the reliability of organ‐on‐a‐chip for pharmacological studies	^44^
Fluidically coupled vascularized organ chips: gut/liver/kidney and bone marrow/liver/kidney	Human primary hepatocytes, human primary LSECs, primary human RPTECs, primary human GMVECs, Caco‐2 BBe, HUVECs, primary human CD34+ cells, stromal cells	Nicotine, cisplatin	In vitro‐in vivo translation drug pharmacokinetics (PK) and pharmacodynamics (PD) parameters Modelling of first‐pass drug metabolism Prediction of drug absorption, distribution, metabolism, excretion and toxicity	^56^

Abbreviations: ES cell, embryonic stem cell; GECs, glomerular endothelial cells; GMVECs, glomerular microvascular endothelial cells; HHSTeC, human hepatic stellate cells; H‐InMyoFib, human intestinal myofibroblasts; hiPSC, human‐induced pluripotent stem cells; HUVEC, human umbilical vein endothelial cells; LSECs, liver sinusoidal endothelial cells; NHBE, normal human bronchial epithelial; NPCs, neural progenitor cells; RPTECs, renal proximal tubule epithelial cells; tHESCs, hERT (human telomerase reverse transcriptase)‐immortalized human endometrial stromal cells.

^a^ Commercially available platforms.

Among human organs, the kidney carries out critical metabolic and endocrine functions and has an important role in xenobiotic clearance, waste excretion, and fluid and electrolyte reabsorption.^49^, ^50^ Therefore, it is particularly vulnerable to drug‐induced toxicity. Liver metabolism actively contributes to the changes in nephrotoxicity, releasing toxic/nontoxic drug metabolites and changing compound bioavailability and plasma concentrations.^49^, ^50^ Drug‐induced nephrotoxicity being associated with a high proportion of adverse effects and drug withdrawal, some multi‐organ‐on‐a‐chip studies focused on developing coupled liver/kidney chips.^49^, ^50^ Li et al. used an integrated liver‐kidney chip device to assess hepatic metabolism‐dependent nephrotoxicity of ifosfamide (IFO) and verapamil (VER). Upon metabolism in the liver, IFO is converted into toxic metabolites, while renal toxicity and biological availability of VER are significantly reduced. These effects were successfully demonstrated in the described model by comparing cell viability, LDH leakage and renal permeability parameters in single‐organ chips with the integrated liver‐kidney chip.^49^ Similarly, another study developed a two‐organ‐chip integrating liver spheroids and proximal tubule barriers.^50^ The study was able to investigate the effects of repeated‐dose administration of Cyclosporine A (CsA) as well as its combination with rifampicin (RFP) simultaneously on two organs.^50^ The importance of integrating liver spheroids was that RFP is able to induce hepatic enzymes and transporter activity to mitigate the toxicity of CsA. Moreover, the platform was identified as promising in detecting morphology, histopathology, drug metabolism and noninvasive toxicity biomarkers in pharmacological studies.

**FIGURE 2:**
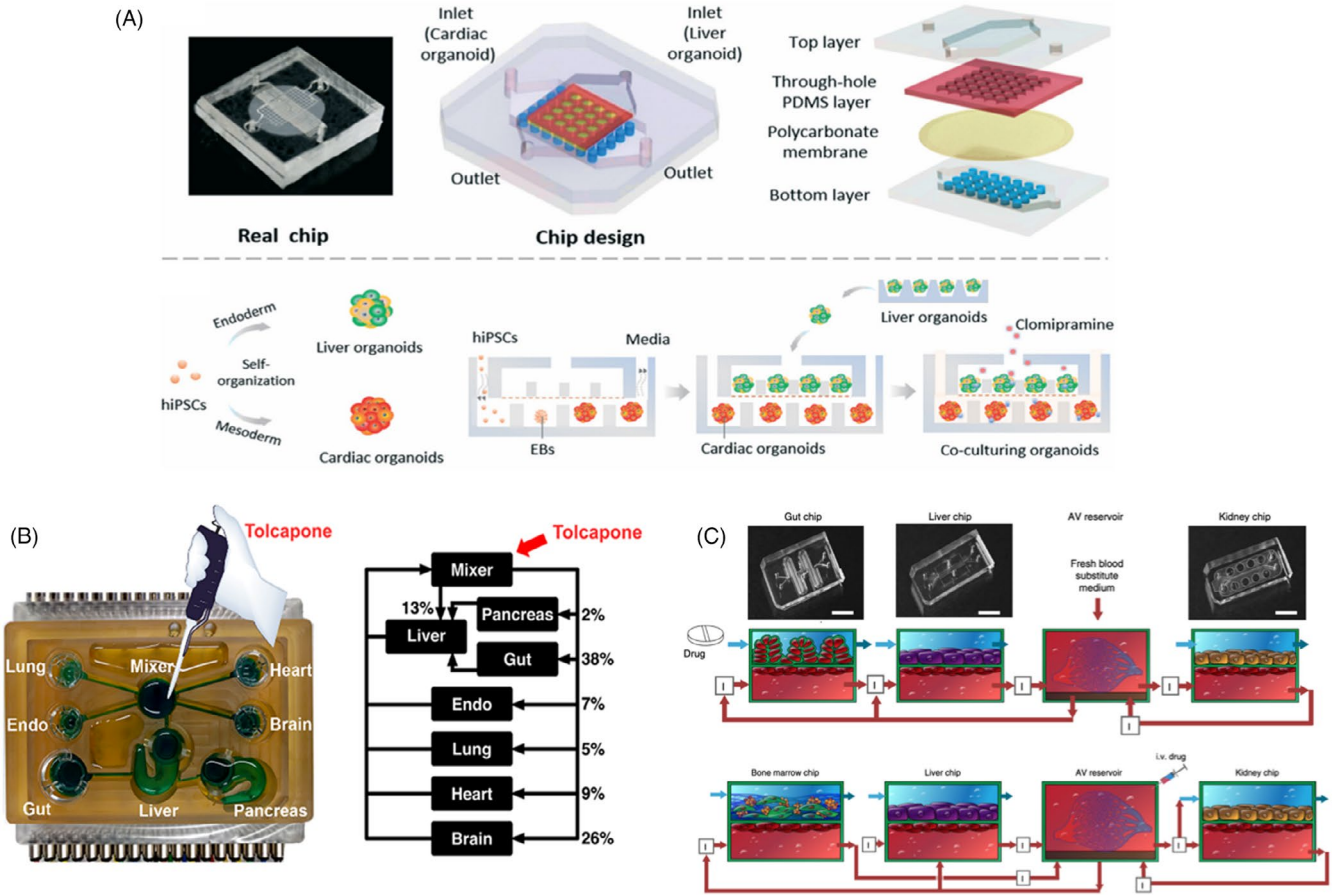
Multi‐organ platforms integrating Liver‐on‐a‐Chip devices (A) Liver‐heart organoids‐on‐chip device. Design and schematic overview of the experimental procedures. Reproduced from Ref. [52], with permission of The Royal Society of Chemistry. (B) Human‐on‐a‐chip system. Reprinted from Ref. [60], with permission from American Chemical Society, Copyright 2019. (C) First‐pass multi‐organ chip system. Reproduced from Ref. [56], with the permission from Springer Nature, Copyright 2020

Similar to investigating nephrotoxicity, cardiotoxicity is also of importance due to many known cases of drug withdrawals from the market caused by cardiovascular side effects and cardiotoxicity.^51^, ^52^ A heart‐liver organ‐on‐a‐chip system would allow studying drug cardiotoxicity induced by hepatic metabolism and identifying effective yet safe metabolites of toxic compounds.^51^ As in nephrotoxicity studies, the presence of a liver component in coupled organs‐on‐a‐chip was able to induce cardiotoxic effects of cyclophosphamide, while alleviating terfenadine toxicity, as evidenced by electrical and mechanical activity of the heart component containing iPSC‐derived cardiomyocytes.^51^ The pharmacokinetic studies employing quantification of CYP activities and metabolite tracking by HPLC‐MS were conducted as well. Another study by Yin et al.^52^ was able to similarly demonstrate hepatic metabolism‐dependent cardiotoxicity of antidepressant drug clomipramine using a hiPSC‐derived multi‐organoid‐on‐chip system (Figure 2A).

The respiratory system is directly exposed to various aerosols and airborne pathogens, and also serves as a route of administration for inhalation medications.^53^ Although initially absorbed in the lung and partially metabolized by lung CYP enzymes, inhaled compounds undergo major metabolic processes in the liver, which gives rise to new metabolites.^53^, ^54^ Therefore, reported lung/liver‐on‐a‐chip platforms focus on the investigation of the toxicity of inhaled substances and the protective effect of the liver granted by the imitation of organ crosstalk.^53^, ^54^ Bovard and colleagues demonstrated a lung/liver‐on‐a‐chip integrating normal human bronchial epithelial (NHBE) cells on an air‐liquid interface (ALI) and HepaRG spheroids. In this study, the toxicity of carcinogenic aflatoxin B1 (AFB1) was reduced in the presence of HepaRG spheroids. Schimek et al. used a HUMIMIC Chip3plus (TissUse GmbH, Berlin, Germany) platform to co‐culture bronchial MucilAir culture and HepaRG + primary human hepatic stellate cells (HHSTeC) spheroids and attained similar results. The protective activity of HepaRG spheroids is attributed to their ability to convert AFB1 to aflatoxin Q1 (AFG1), a less toxic metabolite, while NHBE ALI culture is only able to metabolize AFB1 into two toxic metabolites AFB1‐8,9‐epoxide and aflatoxin M1‐8,9‐epoxide.^53^, ^54^ However, neither of the studies quantified the presence of parent drug and its metabolites; therefore, this conclusion is not experimentally verified.

Drug metabolism and toxicity evaluation can be improved by modelling enterohepatic circulation (circulation of drugs/metabolites between liver and small intestine) as well as first‐pass metabolism, which can be achieved by combining intestine‐ and liver‐on‐a‐chip systems.^55^, ^56^ Moreover, enterocytes with their active influx and efflux transporters and metabolic enzymes interfere with drug absorption, while absorbed compounds and their metabolites further travel to the liver through the portal vein.^56^, ^57^ These compounds and metabolites may cause damage on the level of the small intestine, as well as further on after transportation to the liver.^56^ Several studies investigating these mechanisms developed intestine/liver microphysiological systems.^55^, ^56^, ^57^ Marin and colleagues demonstrate intestine/liver Two‐Organ‐Chip platform to imitate intravenous and oral administration of acetaminophen, as well as the drug's pharmacokinetic characterization. Dynamic conditions in this 2‐OC were able to reproduce absorption (intestine) and metabolism (liver) phases for APAP, although slower than in vivo.^57^ It was also noted that the liver component of the platform was more sensitive to APAP toxicity. This was attributed to the fact that although the drug is absorbed in the intestine, the metabolism into toxic metabolites happens in the liver. Hepatic cytotoxicity was the result of the production of metabolite N‐acetyl‐*p*‐benzoquinone imine (NAPQI), glutathione depletion caused by which was experimentally proven.^57^ Another group successfully recapitulated the first‐pass metabolism using Intestine‐Liver‐On‐Chip (InLiver‐OC).^55^ In this model, the protective role of the intestine component on hepatic damage was verified by lower lipid drop formation, reduced ROS expression and overall functionality of the liver component in comparison with single Liver‐OC.^55^ Although the compound employed in this investigation was ethanol, it is suggested that the model has the potential to imitate the first‐pass metabolism of drugs and other xenobiotics. Chen et al. also propose a pumpless GI tract‐liver system integrating primary human intestinal epithelial cells (hIECs) and HepG2 C3A liver cells, which due to increased CYP activity of a liver component, tight junctions with authentic transepithelial electric resistance (TEER) values, and good barrier functionality for GI component, as well as the easy operation would serve as an improved tool for drug toxicity studies.

Another domain of interest is the toxicity and metabolism assessment of anticancer therapies due to their inherent toxicity, narrow therapeutic index and the high likelihood of a combination of anticancer therapies with medication for other conditions or prescription of additional anticancer therapies.^40^ McAleer et al.^58^ describe a pumpless reconfigurable multi‐organ‐on‐a‐chip for the investigation of on‐target efficacy and off‐target toxicity of anticancer drugs and their metabolites. Primary human hepatocytes were co‐cultured in this platform either with cancer‐derived bone marrow cell lines or with vulva cancer line, breast cancer line and iPSC‐derived cardiomyocytes. In the first case, the system was treated with imatinib or diclofenac, where imatinib was able to target cancer cell lines with a minimum effect on the liver. Diclofenac, however, was chosen to demonstrate off‐target hepatotoxicity, which was successfully demonstrated by the experimental data.^58^ Moreover, the study was able to detect CYP 3A4 and CYP 2C9 induction not previously described in the literature. The second configuration mainly focused on demonstrating off‐target cardiotoxicity of tamoxifen and verapamil but also confirmed the role of the liver in metabolizing tamoxifen to produce its active metabolite 4‐hydroxytamoxifen. Miller et al.^59^ proposed a multi‐organ microfluidic platform with a breathable lung chamber to assess and compare curcumin intravenous delivery and inhalation therapy for breast cancer treatment. The platform included liver, lung, breast cancer components and liver components consisting of HepG2 C3A cells.^59^ Via urea synthesis assays, the study was able to demonstrate that inhalation therapy could induce higher metabolism rates and increase urea production.^59^

Several studies demonstrate more complex MPSs integrating three, six or seven organs.^56^, ^60^, ^61^, ^62^, ^63^, ^64^ Wang and colleagues employed a multi‐organ human‐on‐a‐chip integrating seven interacting MPSs: brain, pancreas, liver, lung, heart, gut, endometrium and a mixer chamber emulating systemic circulation (Figure 2B). The platform was used to create an extensive metabolite profile and metabolomics of tolcapone, a drug used in Parkinson's disease management. The study identified 12 metabolites and reactions responsible for their generation.^60^ This suggests that such multi‐organ platforms involving a liver component are suitable for comprehensive drug metabolism investigations and have the potential in the domain of analytical chemistry. Another group reported two studies involving 3‐organoid (liver, heart, lung) and 6‐organoid (liver, heart, lung, endothelium, brain, testis) multi‐tissue organ‐on‐a‐chip platforms.^63^, ^64^ In these investigations, the panel of FDA‐recalled drugs, as well as anticancer drugs capecitabine and ifosfamide, were screened. Among ten drugs recalled from the market, bromfenac, tienilic acid and troglitazone demonstrated hepatotoxicity.^64^ In the same study, it was demonstrated that the platforms without liver organoids present were not able to metabolize capecitabine and cyclophosphamide and therefore escaped cytotoxicity caused by their metabolites. Similarly in the second study, there was a decrease in viability of lung and heart organoids in the 3‐tissue system involving the liver upon administration of capecitabine, which proves that liver organoid metabolized the drug into its toxic active metabolite 5‐fluorouracil (5‐FU).^63^ In the 6‐tissue system, ifosfamide administration results in neurotoxicity (reduced brain organoid viability) in the presence of liver organoid, again proving that the drug was metabolized into its toxic metabolite chloracetaldehyde due to hepatic P450 metabolic activity. Herland et al. employed multi‐organ‐chip emulating the first‐pass metabolism to create an in silico drug metabolism pharmacokinetic model to perform an in vitro/in vivo translation (IVIVT). The study created two‐channel lung‐liver‐kidney and bone marrow‐liver‐kidney chips fluidically coupled through vascular endothelium‐lined channels and incorporating arteriovenous (AV) reservoir mimicking systemic circulation (Figure 2C). The PK profile of orally administered nicotine and PK/PD parameters for intravenous administration of cisplatin was modelled employing the system. The recreation of endothelial‐parenchymal tissue interface due to the presence of two channels, lined by organ‐specific parenchymal cells and vascular endothelium, as well as successful in silico modulation of drug absorption by poly‐dimethylsiloxane polymer and organs not incorporated into the chip, allowed for an IVIVT approach highly optimized for drug PK/PD modelling.^56^ Overall, these studies propose a more physiologically relevant platform that models a complex and integrated response to drug administration generated by organ crosstalk. The potential of organ‐on‐a‐chip technology itself is promising, and it is estimated to reduce R&D costs per new drug by 10%‐26%.^65^ However, despite the much progress in the development of the individual and multi‐organ platforms, the platforms need to prove reproducible, robust and capable of high throughput screening for the technology to replace traditional drug development methods.

## 6 CONCLUSION

Microphysiological platforms have the potential of creating more effective alternatives to conventional methods in drug development. Among such platforms, liver‐on‐a‐chip (LOC) devices as well as multi‐tissue organ‐on‐a‐chip platforms offer new approaches in predicting drug toxicity during preclinical testing. LOC and multi‐organ LOC models have numerous advantages over two‐dimensional or 3D structure such as more accurate simulation of delivery and penetration of drug compounds, capability to be integrated with analytics and sensors.

Due to their ability to generate the human‐relevant response that can potentially better predict the toxicity of a tested drug compound than traditional in vitro methods, liver‐on‐a‐chip (LOC) platforms with different complexities, throughputs, and cell sources have been developed and used in research and industry (Roche, Takeda Pharmaceutical, AstraZeneca).^66^ LOC has numerous advantages over two‐dimensional systems such as more accurate simulation of delivery and penetration of drug compounds, capability to be integrated with analytics and sensors. Additionally, the continuous flow in LOCs allows monitoring the changes in drug concentration at pre‐defined time intervals, mimicking effectively oral dose exposures.

Liver‐on‐a‐chip integration into the multi‐organ platforms enables to produce even more physiologically relevant models for drug toxicity assessment and evaluate off‐target drug toxicity in parallel with hepatotoxicity studies. The growth and advancement of fabrication tools and techniques have had a positive impact on the complexity of developed LOC models (OrganoPlate Livertox, Liver‐Chip, HUMIMIC devices) and multi‐organ platforms. Novel easy‐to‐build LOC systems are compatible with automated systems^38^ and allow noninvasive biomarker assessment.^50^ These platforms are comparable to traditional 2D platforms in terms of adaptability to high throughput screening settings and have an upper hand over the low‐throughput animal models.^38^ In particular, pumpless or rocker platform‐based systems have the potential for high throughput applications due to their low costs and ability of a rocker platform to sustain a number of LOC units simultaneously.^62^

However, despite the advancements of LOC models, they have limitations in reproducibility of results and difficulty in mapping the obtained results to existing measurement techniques. For instance, primary human hepatocytes, through the most physiologically relevant cell type, demonstrate high lot to lot and donor‐to‐donor variation and potential to de‐differentiate, which has a negative effect on reproducibility.^38^, ^41^ Moreover, since current microscopes are not suited for the organ‐on‐a‐chip applications due to these platforms’ design and set‐up, image acquisition and quantification require the development of new methods and technologies, especially for high throughput settings.^67^ Overcoming the current limitations and improvements in LOC systems is required to achieve acceptance by regulatory bodies and adoption of this technology.

## CONFLICTS OF INTERESTS

The authors have no conflicts of interest to declare.

## AUTHOR CONTRIBUTIONS

GK and AK wrote and edited the manuscript.

## Data Availability

All data used to support the findings of this study are included within the article.
